# The Emotion Authenticity Recognition (EAR) test: normative data of an innovative test using dynamic emotional stimuli to evaluate the ability to recognize the authenticity of emotions expressed by faces

**DOI:** 10.1007/s10072-024-07689-0

**Published:** 2024-07-18

**Authors:** Cristina Scarpazza, Chiara Gramegna, Cristiano Costa, Rachele Pezzetta, Maria Cristina Saetti, Alice Naomi Preti, Teresa Difonzo, Stefano Zago, Nadia Bolognini

**Affiliations:** 1 Department of General Psychology, University of Padova, Via Venezia 8, Padova, PD Italy; 2 IRCCS S Camillo Hospital, Venezia, Italy; 3 Ph.D. Program in Neuroscience, School of Medicine and Surgery, University of Milano-Bicocca, Monza, Italy; 4 Department of Psychology, University of Milano-Bicocca, Milan, Italy; 5 Padova Neuroscience Center, University of Padova, Padova, Italy; 6 Neurology Unit, IRCCS Fondazione Ca’ Granda Ospedale Maggiore Policlinico, Milan, Italy; 7 Department of Pathophysiology and Transplantation, University of Milan, Milan, Italy; 8 Neurology Unit, Foundation IRCCS Ca’ Granda Hospital Maggiore Policlinico, Milano, Italy; 9 Laboratory of Neuropsychology, Department of Neurorehabilitation Sciences, IRCCS Istituto Auxologico Italiano, Milano, Italy

**Keywords:** Emotion authenticity recognition, Normative values, Emotions, Authenticity perception, Neuropsychological tests

## Abstract

Despite research has massively focused on how emotions conveyed by faces are perceived, the perception of emotions’ authenticity is a topic that has been surprisingly overlooked. Here, we present the Emotion Authenticity Recognition (EAR) test, a test specifically developed using dynamic stimuli depicting authentic and posed emotions to evaluate the ability of individuals to correctly identify an emotion (emotion recognition index, ER Index) and classify its authenticity (authenticity recognition index (EA Index). The EAR test has been validated on 522 healthy participants and normative values are provided. Correlations with demographic characteristics, empathy and general cognitive status have been obtained revealing that both indices are negatively correlated with age, and positively with education, cognitive status and different facets of empathy. The EAR test offers a new ecological test to assess the ability to detect emotion authenticity that allow to explore the eventual social cognitive deficit even in patients otherwise cognitively intact.

## Introduction

Social cognition refers to the processing of social information that underlies abilities such as the detection of other’s emotions and the appropriate response to these emotions. As human beings are social creatures, these abilities are considered pivotal for effective social interactions and mental wellbeing [1]. Research has so far investigated many social cognitive abilities, that could be grouped into four categories: theory of mind (ToM), emotional empathy, emotion perception and social behaviour [2].

Among the four delineated categories of social cognition, clinical neuropsychologists have focused particularly on how we correctly identify emotions conveyed by faces, whose expressions are innate and universally recognized across cultures [3]. This subject has gained predominant attention within the realms of neuroscientific and neuropsychological research (e.g., [4–8]), leading to the understanding of the neural circuits involved in the processing of emotional faces (e.g., [9–12], of dynamic emotional faces (e.g., [12], of the central role of amygdala and insula for fear and disgust processing, respectively (e.g., [13, 14], of the role of task and stimuli characteristics on brain responses (e.g., [15], of the psychophysiological reactions to other’s emotions (e.g. [16–18] and so on.

This extensive knowledge clearly contributed to the massive clinical translation of the research findings that has been made possible thanks to the creation of psychometric tests to evaluate individuals’ ability to correctly recognize emotions expressed by others. Examples of these tests include, for instance, the Ekman 60 faces test emotion labelling and emotion discrimination [19, 20], the Reading the Mind in the Eye [21], the FACE test [22], the Geneva Emotion Recognition test [23]. Recently, neuropsychological batteries specifically designed to evaluate social cognition have been developed and they include tests to assess emotion recognition capabilities, among which the Florida Affect Battery [24], the Comprehensive affect testing system [25], the EMOTICOM [26], and one battery specifically created to measure recognition of facial expression of emotions [27].

The clinical application of these instruments allowed neuropsychologists to clarify that emotion recognition deficits appear to be a core cognitive phenotype of many brain-based disorders [28], being prevalent in neurological (e.g., [29–35] and psychiatric diseases (e.g., [36–39], but they also represent a core marker of developmental disorders (e.g., [40, 41], personality disorders [42–44] and personality traits [44–47].

Despite the significant advancements in understanding emotions’ recognition from faces and its impairment in brain-based disorders [2, 28, 48–51], a failure to engage and reciprocate socially, even when obvious social cues are given, could not derive solely by difficulties in the identification of emotions. Indeed, emotions identification can be considered sometimes an easy task: when the emotion is unambiguous (i.e., sufficiently intense and not blended, [52]), for example, recognizing that a smile means happiness and not anger can be a straightforward task. In this view, it has been postulated that the rational recognition of emotions can also rely on semantic knowledge [53]. In other words, the fact that the concomitant presence of upwardly pulled lip corners and raised cheeks represents a smile can be guided by conceptual knowledge of emotions. This idea is supported by data on patients with semantic variant of frontotemporal dementia, where semantic deficits are demonstrated to play a critical role in facial emotion recognition [53].

However, one of the most important aspects of communication and social interaction lies in the perceived authenticity of the expressed emotion, as influenced by genuine experience [54, 55], termed as “event-elicited” [56]. Indeed, impairment in social behaviour often arise as a direct consequence of emotional misinterpretation: even when people are able to adequately understand which emotion is expressed on other’s face, an anomalous behaviour in social situations could emerge from the failure in the identification of the authenticity of emotions expressed by others. Certainly, emotional expressions do not always reflect our true feelings [57]. Fear may arise from encountering a real snake, or sadness from the loss of a loved one. In this case emotions may be generated by genuine experiences [54, 55, 58, 59]. Conversely, individuals may deliberately simulate emotions without a corresponding genuine context in order to gain strategic advantages [60, 61].

This is extremely relevant for effective social interactions: for instance, a smile perceived as genuine might promote social interaction, while a smile perceived as not genuine might promote avoidance. Similarly, genuine anger might promote avoidance, while posed anger might not. Because mistaking posed emotions for genuine can result in negative outcomes for the perceiver, any impairment in the ability to distinguish authentic from posed emotions might help explain why individuals suffering from psychiatric and neurological disorders often find it difficult to engage socially [2, 62]. Furthermore, research has shown that distortion or misinterpretation of social cues can result in the generation of inappropriate social responses, such as aggressiveness or violence, especially in ambiguous situations [63]. Despite individuals can try to rely on semantic knowledge to overcome their difficulties [53], this might be extremely difficult, or even impossible, when they are asked to evaluate such a subtle feature, as the authenticity of emotion. Thus, determining whether individuals exhibit deficits in discerning emotional authenticity is extremely relevant both for a comprehensive understanding of individuals social abilities.

Despite the clear importance of the correct identification of emotions authenticity, so far this topic has been surprisingly neglected, remaining largely unexplored: virtually nothing is known on how we perceive and categorize emotion’s authenticity and about whether this ability is impaired in neurological and psychiatric diseases. Yet, the emotional stimuli classically used in research, as well as implemented in clinical tests, depict faces where emotions are posed [19]. Some dataset including both authentic and posed emotions are now available for research (for a review see [64]), but they have drawbacks (e.g., included only happiness, using only 2D images, absence of validation) limiting their usability in clinical and research settings. Using 2D static stimuli is not only poses problems of ecological validity, but also limits our understanding of facial cues. By contrast dynamic facial stimuli c a richer and more realistic representation of emotional expressions, which facilitate higher emotional judgement (i.e. intensity and arousal) and the accuracy of emotion recognition [65, 66].

The aforementioned issue is of particular relevance for neuropsychologists, as the few available neuroscientific research supports the idea that authentic and posed emotions may involve distinct neuro-cognitive mechanisms. First, authentic and posed expressions differ in terms of temporal and morphologic features [52, 67, 68], where authentic emotions can occur in millisecond (e.g., micro-expressions) and are often less intense and more subtle than posed expressions [69], while posed emotions are often prototypical and very intense [4]. Second, genuine and posed emotions differ in terms of neural bases. Evidence from a fMRI study [70] suggests that observing genuine emotions enhances the activity of the left medial superior frontal gyrus and the middle cingulate cortex bilaterally, as compared to observing fake emotions. Unfortunately, the authors employed only 2D static expressions, in turn limiting the perceived authenticity of the emotions by the observers. A second fMRI study revealed that the perception of authentic vocal emotion expression enhances emotion recognition and the activity of the ToM network [71].

Taken together, current evidence supports the idea that authentic and posed emotions can be processed by a two at least partially distinct neural networks, paving the way for the hypothesis that the ability to recognize emotion’s authenticity can be selectively damaged by brain disorders. Little is still known on this topic, as so far research focused on authentic smile only. Early research revealed that Duchenne smile (i.e., a smile characterized by the contraction of the orbicularis oculi, considered to be the marker for authentic smile) is considered more authentic than non-Duchenne smile [72], while later research supporting the idea that Duchenne smiles are not indicative of felt emotions as they can be deliberately displayed [73]. Additionally, both autism [74] and psychopathy [75, 76] are found to affect the ability to correctly identify an emotion as genuine or not. However, despite these promising results, the paradigms used to assess such ability remain experimental and they lack ecological validity (i.e., static images rather than 3D video).

For all these reasons, we have developed the Emotion Authenticity Recognition (EAR) test, a new test specifically created to evaluate individuals’ ability to discriminate between authentic and posed dynamic emotions. The EAR test was created to address limitations present in the available neuropsychological assessment aiming at evaluating emotion recognition: first, it includes stimuli depicting authentic (i.e., the emotions were elicited in the participants) and posed (i.e., the participants were asked to pose each emotion) emotions, taken from the Padova Emotional Dataset of Facial Expressions (PEDFE), a unique dataset of genuine and posed emotions [77]; second, it comprises dynamic emotional stimuli (i.e. short videoclips) in order to provide the observers with important information regarding the dynamic of facial expressions, helpful to distinguish genuine from posed emotions [52, 68, 78].

The present study aims at validating and deriving normative data of the EAR test in a large sample (*n* = 522) of neurologically and psychiatrically healthy adults for favoring its adoption in clinical practice and experimental research. To this aim, we also explored the association between the ability of recognizing emotions, and their authenticity, with empathic abilities and global cognitive functioning; the latter aspect only in participants aged > 50 years.

## Materials and methods

### Participants

In line with previous normative studies [79, 80], the minimum sample size was set at *N* = 286 by means of a power analysis (*α* = 0.05; 1-*β* = 0.9; *f*^2^ = 0.05) for multiple linear regression (*df*_numerator_ = 3) analyses [81] via the R package pwr [82].

We recruited a sample of 522 Italian adults (306 women and 216 men) of different ages (mean: 39.97 ± standard deviation 14.64 years; range: 17–88 years) and educational level (mean: 15.12 ± 3.08 years, range: 5–24 years), most of them living in northern Italy. Inclusion criteria were: (1) absence of previous/current history of neurological, psychiatric and/or severe general medical condition; (2) normal or corrected-to-normal vision and hearing; (3) performance above cut-off score (equivalent score > 0) at the Montreal Cognitive Assessment (Nasreddine et al., 2005; Italian normative data by [83], which was administered only to 148 participants who aged ≥ 50 years.

Sample stratification and participants’ demographic data are reported in Table 1.

**Table 1:** Sample stratification for age, education, and sex

	M F	Age						
Education		**25≤**	**26–35**	**36–45**	**46–55**	**56–65**	**66–75**	**≥ 76**
5≤		0/0	0/0	0/0	0/0	0/1	0/0	0/1
6–13		11/14	20/13	19/24	23/29	27/28	2/4	3/2
14–16		19/37	11/14	4/10	9/5	0/5	0/0	0/0
≥ 17		15/38	17/27	10/16	12/23	11/13	3/0	0/0

Cells show male/female ratio for each co-occurrence

Individuals took part in this study on a voluntary basis, after having provided their written informed consent. The study was approved by the Ethics Committee of the University of Padova (Protocol Number: 3954), in compliance with the guidelines of the Helsinki Declaration (1975).

### Emotion authenticity recognition (EAR) test

The Emotion Authenticity Recognition (EAR) Test consists in the presentation of sixty brief videos, selected from the Padova Emotional Dataset of Facial Expressions (PEDFE; [77], a validated dataset including the dynamic emotional expression of the six universal emotions (happiness, surprise, sadness, anger, fear, and disgust) from 56 participants. Critically, participants’ emotional expressions could be authentic (i.e., the emotional status was really induced in the participants using an experimental procedure, *n* = 707) or posed (i.e., asking the participant to pose a specific emotion, *n* = 751).

For the current study, 60 dynamic stimuli were selected from the PEDFE dataset: 10 for each universal emotion, of which 5 depicting authentic emotions, and 5 depicting posed emotions (for a total of 30 authentic and 30 posed emotional expressions). Among the 1458 stimuli present in the PEDFE dataset, we incorporated into the EAR test those stimuli that exhibited the highest accuracy in recognizing emotions and correctly identifying the authenticity categorization within the normative sample, in order to attain a ceiling effect. The mean percentage of accuracy rate was 93.9% for emotion and 84.4% for authenticity recognition in the original validation sample.

Selected stimuli were 3.05 s long in average (standard deviation: 2.77, range: 0.79–13.66). The videos length does not differ between authentic and not authentic emotions (two independent sample t test *p* = .26) but differs between emotions, as some emotions are expressed very quickly (for instance fear, mean 1.69 s), while other emotions are very slow (for instance sadness, mean 7.91 s), as described in the original publication [77].

**Fig. 1:**
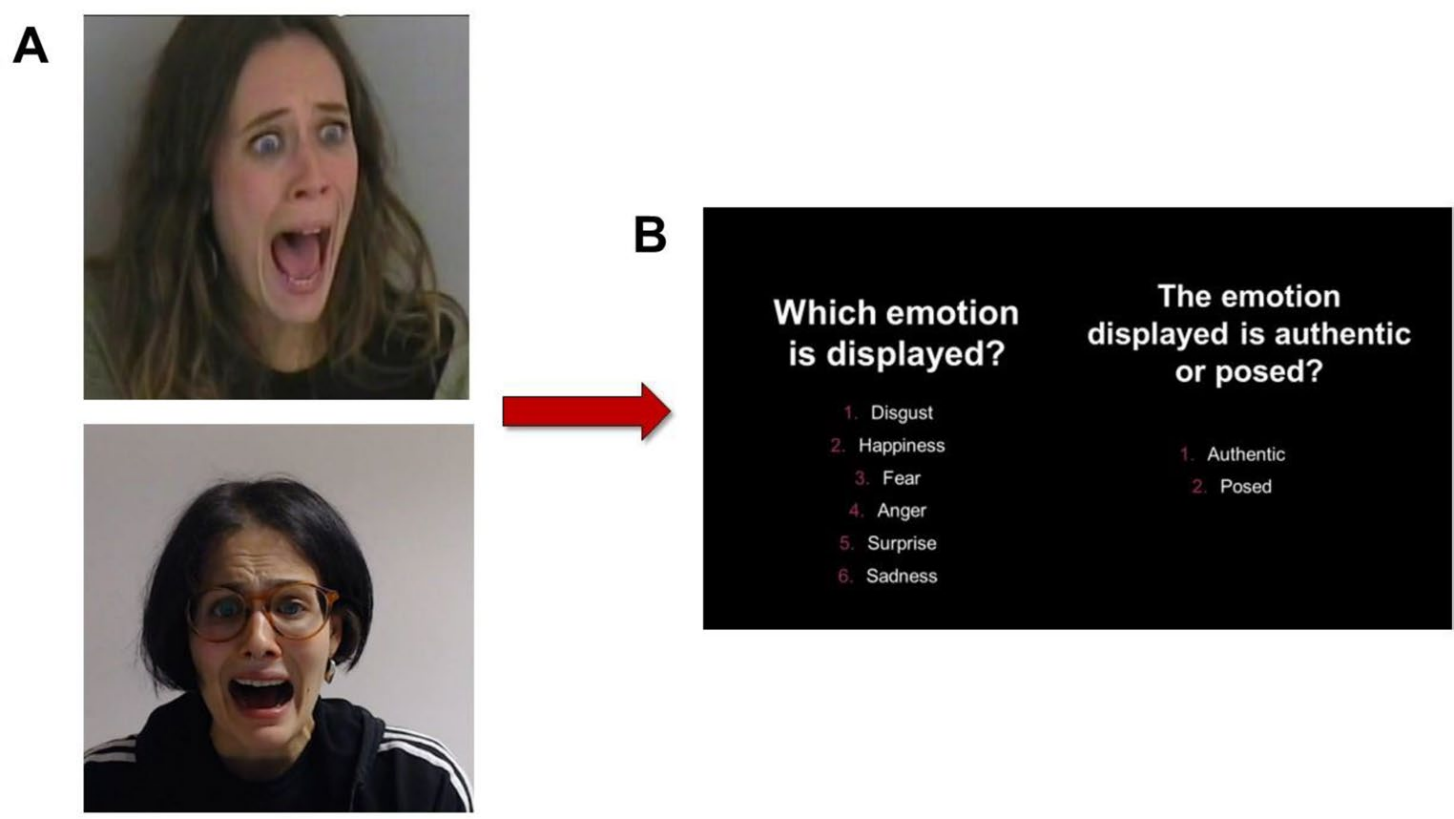
Experimental procedure of the Emotion Recognition Test: (**a**) emotion presentation (e.g., fear), genuine (above) and posed (below); (**b**) experimental questions

In the current study, volunteers were asked to observe the short videos depicting authentic and not authentic emotions and to: (1) identify the emotion conveyed by faces by choosing it among 6 alternatives, and (2) classify emotions as authentic or posed (see Fig. 1). One point is attributed for each correct identification or classification. Two indices can be obtained: (1) an Emotion Recognition Index (ER Index) and (2) an Emotion Authenticity recognition index (EA Index). The maximum score for each of the two index is 60.

### Experimental procedure

Participants were tested in a quiet room by one interviewer. In addition to the EAR test, the MoCA [83, 84] and the Interpersonal Reactivity Index (IRI; [85, 86] were administered.

The MoCA is a psychometrically robust screening test for detecting cognitive decline. It takes about 10 min to administer and comprises 30 items assessing multiple cognitive domains: short-term and working memory, visuospatial abilities, executive functioning, attention, and language.

The IRI is a 28-item self-report questionnaire to measure empathy through four subscales of 7-item each covering different facets of empathy: Empathic Concern, Personal Distress, which together reflect the affective side of empathy, and Perspective Taking and Fantasy measuring cognitive empathy. The test requires to report the extent to which each of the 28 statements describes oneself on a 5-point Likert scale ranging from 0 (“Does not describe me well”) to 4 (“Describes me very well”). Total score ranges 0–112, with higher scores indicating higher level of empathy. IRI compilation requires about 10 min.

### Statistical analyses

Normality checks on raw data (number of correct responses) were performed by assessing skewness and kurtosis values (judged as abnormal if ≥|1| and |3|, respectively; [87], as well as by visually inspecting histograms and quantile-quantile plots [88].

In order to derive normative values for the EAR test, the Equivalent Scores (Ess) method was adopted [89, 90]. To this aim, raw scores were adjusted for significant intervening background predictors (or their transforms) via regression-based equations. The cut-off was then identified by computing outer and inner tolerance limits (oTL and iTL, respectively). Adjusted scores (Ass) were standardized into a 5-level quasi-continuous scale: ES = 0 (Ass ≤ oTL; “abnormal”); ES = 4 (Ass > Mdn; “normal”); ES = 1, 2, and 3 (oTL < Ass ≤ Mdn; respectively, “borderline,” “low-end normal,” “normal”).

Associations between task performance and IRI (and IRI subscales) and MoCA scores were assessed by means of Pearson’s parametric test.

SPSS 28.0.1 [91] and R 4.2.1 [92] were adopted to run the analyses. TLs and Ess thresholds were calculated according to software solutions provided by Aiello & Depaoli [93].

## Results

Participants’ demographic measures are reported in Table 1, whereas participants’ test scores are summarized in Table 2.

**Table 2:** Participants’ sex, mean age, and mean scores ± standard deviation (score range) for each test

Sex (M/F)	Age (years)	Education (years)	ER Index (*N* = 522)	EA Index (*N* = 522)	MoCA (*N* = 148)	IRI (*N* = 485)
216/306	39.97 ± 14.64 (17–88)	15.12 ± 3.08 (5–24)	51.17 ± 5.13 (31–60)	50.17 ± 5 (33–60)	27.95 ± 2.03 (19–30)	66.4 ± 13.19 (22–104)

EAR = Emotion Authenticity Recognition test; ER Index = Score at the Emotion Recognition subtest; EA Index = Score at the Emotion Authenticity recognition subtest; MoCA = Montreal Cognitive Assessment; IRI = Interpersonal Reactivity Index; N = number of participants

Two separate regression analyses were conducted: the first involved the raw score for categorization of the emotions portrayed (i.e., Emotion Recognition Index, ER Index), while the second concerned the raw score for the identification of authentic vs. posed emotions (Emotion Authenticity recognition Index, EA Index).

EAR scores were negatively associated with age (ER Index *r* = − .498; *p* < .001, EA Index: *r* = − .450; *p* < .001), whereas positively with education (ER Index: *r* = .142; *p* = .001, EA Index: *r* = .161; *p* < .001).

**Fig. 2:**
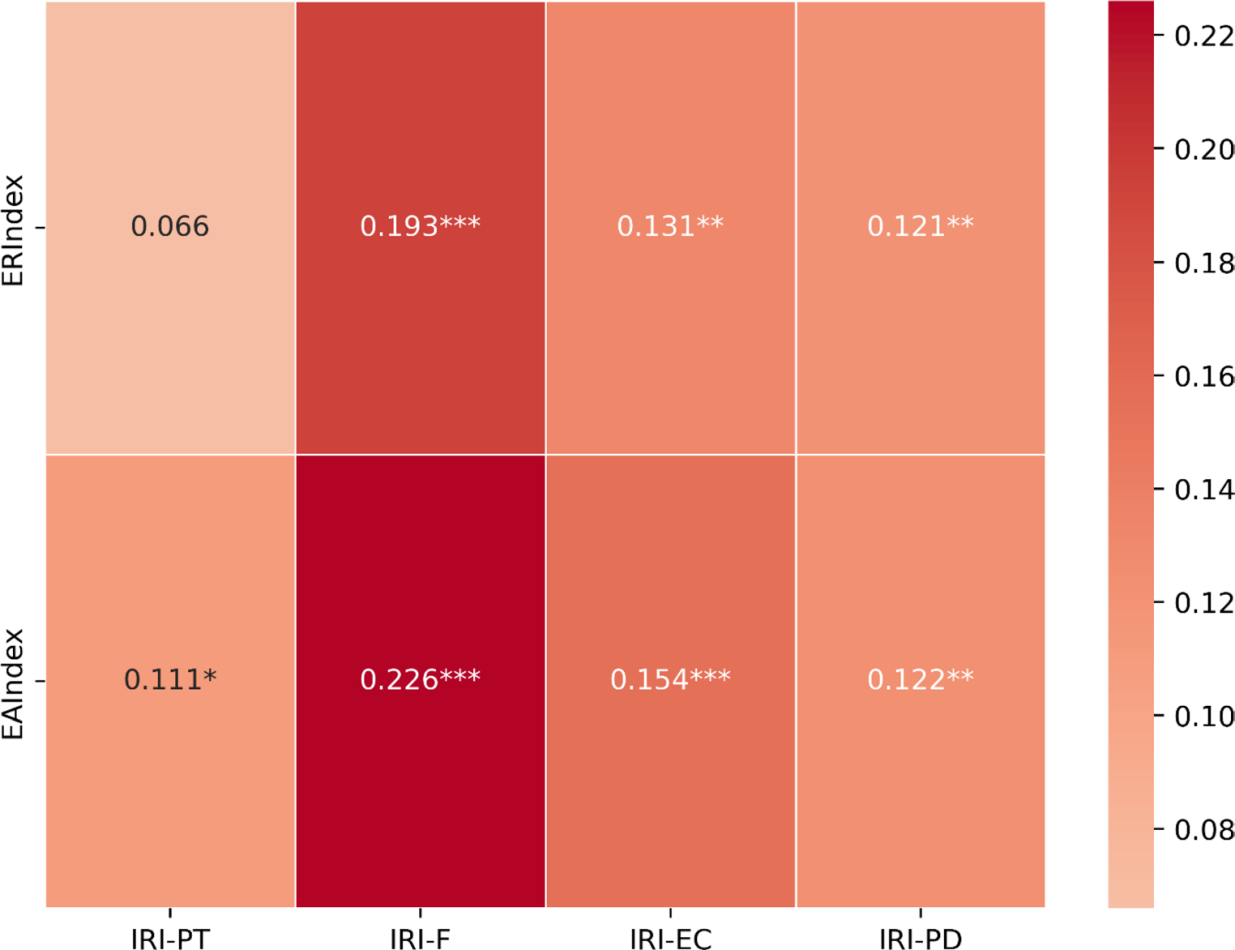
Correlations between ER Index and EA Index scores and IRI subscales. ERIndex = Emotion Recognition Index; EAIndex = Authenticity Recognition Index; IRI = Interpersonal Reactivity Index; PT = Perspective Taking; F = Fantasy; EC = Empathic Concern; PD = Personal Distress. *=*p* values < 0.05; **=*p* values < 0.01; ***=*p* values < 0.001

EAR scores proved to be positively associated with global cognition (MoCA; ER Index: *r* = .450; *p* < .001; EA Index: *r* = .383; *p* < .001) and dispositional empathy (IRI; ER Index: *r* = .189; *p* < .001, EA Index: *r* = .232; *p* < .001) measures. Correlations between ER Index and EA Index and IRI subscales are presented graphically in Fig. 2.

In multiple regression models, transformed age and sex significantly predicted both the ER Index and the EA Index. Adjustment equations and selected correction factors for ER Index and the EA Indexare shown in Tables 3 and 4, respectively. TLs and Ess for both ER Index and the EA Index are reported in Table 5.

**Table 3:** Adjustment grid according to age and sex for the ER index raw accuracy score

	Age												
	25	30	35	40	45	50	55	60	65	70	75	80	85
M	-1.56	-1.01	-0.36	0.40	1.25	2.20	3.25	4.40	5.65	7.0	8.45	10.0	11.65
F	-3.19	-2.64	-1.99	-1.24	-0.39	0.56	1.61	2.76	4.01	5.36	6.81	8.36	10.01

M = male; F = female. Adjusted score = raw accuracy score − 0.002*[(age^2^) – 1811.257] + 0.818 if male; − 0.818 if female. Significant decimals of adjustment factors are displayed

**Table 4:** Adjustment grid according to age and sex for the EA Index raw accuracy score

	Age												
	25	30	35	40	45	50	55	60	65	70	75	80	85
M	-1.95	-1.40	-0.75	-	0.85	1.80	2.85	4.0	5.25	6.60	8.05	9.60	11.25
F	-2.79	-2.24	-1.59	-0.84	0.01	0.96	2.01	3.16	4.41	5.76	7.21	8.76	10.41

M = male; F = female. Adjusted score = raw accuracy score − 0.002*[(age^2^) – 1811.257] + 0.420 if male; − 0.420 if female. Significant decimals of adjustment factors are displayed

**Table 5:** Equivalent scores for the ER index and EA Index adjusted scores

Equivalent scores							
	**oTL**	**iTL**	**0**	**1**	**2**	**3**	**4**
ER Index	42.35	44.71	≤ 42.35	42.36–46.09	46.10-48.87	48.88–51.45	≥ 51.46
EA Index	41.25	43.23	≤ 41.25	41.26–45.09	45.10-47.77	47.78–50.62	≥ 50.63

ER Index = Emotion Recognition index; EA Index = Emotion Authenticity recognition index; oTL = outer tolerance limit; iTL = inner tolerance limit

Participants’ mean recognition scores for each emotion are reported in Table 6. Interestingly, while happiness, surprise, sadness and disgust are recognized with high accuracy (around 9 out of 10 correct recognition), the emotion of fear is the one with the lower correct recognition accuracy (6.54 ± 2.24 out of 10), followed by anger (7.96 ± 1.69 out of 10). See Table 6 for details.

**Table 6:** Participants’ mean score ± standard deviation (score range) for each emotion

Happiness	Surprise	Anger	Fear	Sadness	Disgust
9.29 ± 0.91	9.46 ± 0.87	7.96 ± 1.69	6.54 ± 2.24	9.07 ± 1.28	8.82 ± 1.16
(5–10)	(4–10)	(2–10)	(0–10)	(4–10)	(4–10)

## Discussion

The EAR test represents a novel neuropsychological instrument specifically developed to evaluate the ability of individuals to identify emotions expressed by others as authentic or posed. It comprises of two subtests: one measuring the ability to correctly identify emotions (ER Index), while the EA Index subtest measures the ability to correctly identify the authenticity of emotions. This test is unprecedented, as so far, the ability to discriminate authentic from posed emotions have been largely overlooked. The current study provides normative data and cut-off values for the two EAR subscales: in the Italian population, socio-demographic factors such as age, education and gender influence the EAR scores, requiring the application of correction values in clinical contexts.

Although the purpose of our study was the validation of a new test, deriving normative values in healthy Italian populations, some interesting results emerged that deserve further discussion, and lend themselves to future investigation. Firstly, the present study showed in a large sample of healthy individuals that age and education are both related to both EAR scores, with age showing a higher predictive value. In particular, age is negatively associated with both ER Index and EA Index, meaning that as age increases, the performance decreases at both these sub-tests, while education is positively associated with both indices, meaning that as education increases, the performance at the test increases too. These data support and expand previous results showing that aging is associated with increased difficulties in recognizing emotional expressions [94–101], with gradual decline of emotion recognition across the healthy human lifespan. The novelty of our study is that ageing also diminishes the capacity to correctly discriminate whether an emotion is authentic or not. This can further contribute to explain why older people manifest more difficulty to socially engage [102, 103]. Previous literature also revealed that individuals with a higher educational level perform better on emotion recognition tasks [97, 104, 105]. The current results suggest that higher education contribute to a better performance in emotional authenticity discrimination too (EA score) (refs). It could be argued that growing up in educational environments characterized by intense and diverse social interactions may enhance not only the efficiency of emotion recognition but also the ability to detect signs of truthfulness or deception. This skill development is likely because such abilities also depend on contextual information, including bodily cues and co-occurring social information. These environments provide numerous opportunities for observing and interpreting a wide range of emotional expressions and social signals, thereby refining these critical social cognitive skills [106–111].

Furthermore, EAR scores correlate with both empathy and cognitive efficiency. The correlation with empathy (as measured by means of IRI) is positive, meaning that individuals with a higher self-reported empathy achieved better results both in emotion and in authenticity recognition. Despite this result supports previous literature on emotional recognition [112–115], the correlation between the EA Index and empathy, although significant, is moderate, suggesting that EAR and IRI evaluate different aspects of social cognitive functioning. Interestingly, while the ER Index correlates only with the fantasy subscale of the IRI (ability to put oneself into fictional situations and take the perspective of fictitious characters), in line with previous evidence (e.g., [116, 117], the EA index correlates not only with fantasy but also with perspective taking score, hence this index is associated with both the IRI subscales measuring cognitive empathy, which reflects more advanced capabilities similar to theory of mind (ToM) [2]. This finding suggests that the ability to recognize others’ emotions and their authenticity are different functions that both rely on cognitive empathy, but only the capacity to understand whether an emotion is authentic or not is favoured by the ability to adopt the psychological viewpoint of others, taking into consideration the perspective of others and distinguishing it form one’s own. This finding suggests that perspective taking may help to understand whether the observed emotion is genuine by increasing interpersonal accuracy, allowing us to capture the spontaneity, and therefore truthfulness, of the facial emotions we observe. Shifting attention on others’ faces can significantly increase the ability to detect expressive cues that convey information about their emotions (e.g., [118]; this improvement in decoding others’ emotional states and intentions aids in discerning the authenticity of the emotions being expressed.

The correlation between EAR (both indexes) and cognitive efficiency, as assessed by MoCA in participants over 50 years old is also positive, indicating that individuals with higher general cognitive efficiency also perform better at the EAR test. This makes the EAR a valuable tool to complement the screening of global cognitive status by allowing a quick and reliable assessment of emotion authenticity abilities.

A final result is that fear is the least accurately recognised amongst the six basic emotions. This effect has been repeatedly observed in previous research on emotions (e.g., [119–123], revealing that the errors are not random, but that fear is typically confused with surprise [121, 124, 125]. This is because fear and surprise share a similar muscle configuration making the facial expressions of these emotions visually similar [126–128]. Indeed, literature suggest that the greater the similarity between facial expressions of emotions, the more difficult it is to distinguish them perceptually [129]. Interestingly, the opposite effect (i.e. surprise being confused with fear) is not reported in literature and, congruently, our data revealed that the recognition of surprise is accurate. This can be explained by data supporting that in response to ambiguity, observers tended to bias their categorization responses toward less socially threatening emotions (e.g., “surprise”) [124].

We do believe that the adoption of the EAR test in clinical practice could provide further insight into brain-disorders neuropsychological underpinnings. Indeed, the EAR test provides a unique opportunity to clarify whether patients with neurological and psychiatric disorders tend to misinterpret the authenticity of perceived emotion. In this light, its usefulness is manifold. First, it could promote a better understanding of some symptoms. For instance, it can be postulated that paranoia could sometimes emerge from misinterpretation of other’s emotions as faked, not genuine, thus promoting suspiciousness and social avoidance. Second, it can help to clarify the origin of social impairment: in addition to ongoing psychiatric or neurological symptoms, misperception of the other’s emotional authenticity may play a causal role in the breakdown of interpersonal abilities, leading to difficulties in reciprocate socially and to social impairment, which is one of the most prominent and disabling features in psychiatric and neurological disorders [2, 62]. For example, the inclusion of tests that uses authentic rather than posed stimuli may improve the neuropsychological contribution to differential diagnosis between frontotemporal dementia subtypes, in which patients show difficulties in emotional processing [130, 131]. Third, it can help enhancing the understanding of the link between emotional authenticity perception deficit and violence/aggressiveness, considering previous evidence suggesting that violence can emerge as a consequence of a defective ability in understanding emotions conveyed by faces [63, 132]. In particular, ambiguous contexts seem to be associated with an increased risk of enacting violent behaviour. A difficulty in recognising an emotion as authentic or not creates ambiguity in social situations. Thus, given the negative impact that a misattribution of authenticity might have on the quality of life of individuals, the EAR test represents a reliable and quick instrument for the assessment of patient’s misinterpretation of the genuineness of emotions in everyday clinical practice, with the possibility of planning effective psychological interventions tailored to each patient’s profile. Critically, the EAR test could be applied to the cognitive evaluation of brain-based disorders (i.e., neurologic, psychiatric, developmental, etc.,) with a clear impact of future knowledge emerging from its application within the mental health community. Certainly, these are only hypotheses that should be tested in future studies.

Despite the undeniable advantages of the EAR test, this study is not devoid of limitations. A first drawback is that we did not calculate the convergent validity with other emotion recognition tests. Second, in this study we did not explore the link between the ability to identify emotion authenticity and the ToM. This topic is relevant as ToM is strongly implicated in emotion recognition [133] suggesting it may also be involved in authenticity recognition. This hypothesis is supported by our results revealing a correlation between EA and cognitive empathy. A third important limitation consist in the fact that individuals aged > 65 years are poorly represented. Thus, caution is needed when using the present regression-based adjustments in elderly, limiting the applicability of the EAR test for studies on normal or pathological aging. Further studies are needed to overcome these limitations.

In conclusion, in the current study we presented the validation and normative values of the Emotion Authenticity Recognition test (EAR test), a novel and promising neuropsychological instrument specifically developed to evaluate the ability of individuals to identify emotions expressed by others as authentic or posed. Given its simplicity of administration, as it only requires a laptop for stimuli presentation and involves verbal responses from patients, it can be easily implemented in everyday clinical neuropsychological practice. The EAR test evaluates a subtle social cognitive ability, providing clinicians with a unique opportunity to explore the potential social cognitive deficits even in patients otherwise considered cognitively intact. As this aspect of social cognition has been so far greatly neglected, there is an urgent need to raise awareness on the importance of these difficulties among clinicians, who can extremely benefit from this additional piece of the puzzle regarding the challenging assessment of social cognition in brain-based disorders. Future studies on the clinical usefulness of the EAR test as a cognitive screening test are necessary to explore its validity and sensitivity to identify cognitive deficits in neurological and psychiatric disorders, as well as in the earliest stages of these diseases and in their pre-clinical manifestations.

## Data Availability

All data have been made publicly available at the Open Science Framework (OSF) repository and can be accessed at https://osf.io/gfuqx/.
